# RETRACTION: Antiarthritic Activity of Qi‐Wu Rheumatism Granule (a Chinese Herbal Compound) on Complete Freund’s Adjuvant‐Induced Arthritis in Rats

**DOI:** 10.1155/ecam/9793428

**Published:** 2026-02-13

**Authors:** Evidence-Based Complementary and Alternative Medicine

RETRACTION: Q. Xu, Y. Zhou, R. Zhang, Z. Sun, and L.‐f. Cheng, “Antiarthritic Activity of Qi‐Wu Rheumatism Granule (a Chinese Herbal Compound) on Complete Freund’s Adjuvant‐Induced Arthritis in Rats,” *Evidence-Based Complementary and Alternative Medicine*, 2017, (2017): 1960517, https://doi.org/10.1155/2017/1960517.

The above article, published online on 07 November 2017 in Wiley Online Library (https://wileyonlinelibrary.com), has been retracted by John Wiley & Sons Ltd. The retraction has been agreed due to figure concerns, initially highlighted on PubPeer [1]. On further investigation, the below concerns have been identified:

• In Figure 2:
• The DEX (5 mg/kg) panel is a cropped duplication of Panel A in Figure 2 of [2].
• The QWRG (1.0 g/kg) panel is a cropped duplication of Panel C in Figure 2 of [2].
• The QWRG (4.0 g/kg) panel is a cropped duplication of Panel D in Figure 2 of [2].
• The Control panel is a cropped duplication of Panel G in Figure 7 of [3].
• The AIA group panel is a cropped duplication of Panel C in Figure 7 of [3].
• The QWRG (2.0 g/kg) panel is a cropped duplication of Panel E in Figure 7 of [3].
• In Figure 4, all panels are identical to panels shown in Figure 4 of [2].
• In Figures 5 and 6, all panels except the AIA group are identical to panels shown in Figures 3 and 4 of [4].

After assessment of the concerns and author’s response by the Chief Editor, the results are no longer deemed reliable and the article is retracted.

The authors did not respond to the retraction.

## References

[1] A. belfragei, “Antiarthritic Activity of Qi-Wu Rheumatism Granule (a Chinese Herbal Compound) on Complete Freund’s Adjuvant-Induced Arthritis in Rats,” *PubPeer*, 2025, https://pubpeer.com/publications/46EF5F521372DEAD8F93C6F87CB963#1. 10.1155/2017/1960517 PMC5697382 29238384

[2] Chen Y. , Wang Q. W. , Zuo J. et al., Anti-Arthritic Activity of Ethanol Extract of Claoxylon indicum on Freund’s Complete Adjuvant-Induced Arthritis in Mice, BMC Complementary and Alternative Medicine. (2017) 17, no. 1, 10.1186/s12906-016-1500-7, 2-s2.0-85008394538. PMC5216541 28056924

[3] Wang D. , Hu S. , Zhu J. et al., Angiotensin II Type 2 Receptor Correlates with Therapeutic Effects of Losartan in Rats with Adjuvant-Induced Arthritis, Journal of Cellular and Molecular Medicine. (2013) 17, no. 12, 1577–1587, 10.1111/jcmm.12128, 2-s2.0-84891159115. 24112447 PMC3914644

[4] Xiao X. , Hou H. , Lin V. et al., Probucol Protects Rats From Cardiac Dysfunction Induced by Oxidative Stress Following Cardiopulmonary Resuscitation, Oxidative Medicine and Cellular Longevity. (2017) 2017, 10.1155/2017/1284804, 2-s2.0-85042623728. PMC5682903 29213348

